# Identification of a Goat Intersexuality-Associated Novel Variant Through Genome-Wide Resequencing and Hi-C

**DOI:** 10.3389/fgene.2020.616743

**Published:** 2021-02-09

**Authors:** Guang-Xin E, Dong-Ke Zhou, Zhu-Qing Zheng, Bai-Gao Yang, Xiang-Long Li, Lan-Hui Li, Rong-Yan Zhou, Wen-Hui Nai, Xun-Ping Jiang, Jia-Hua Zhang, Qiong-Hua Hong, Yue-Hui Ma, Ming-Xing Chu, Hui-Jiang Gao, Yong-Ju Zhao, Xing-Hai Duan, Yong-Meng He, Ri-Su Na, Yan-Guo Han, Yan Zeng, Yu Jiang, Yong-Fu Huang

**Affiliations:** ^1^Chongqing Key Laboratory of Forage & Herbivore, Chongqing Engineering Research Centre for Herbivores Resource Protection and Utilization, College of Animal Science and Technology, Southwest University, Chongqing, China; ^2^Key Laboratory of Animal Genetics, Breeding and Reproduction of Shaanxi Province, College of Animal Science and Technology, Northwest A&F University, Yangling, China; ^3^College of Animal Science and Technology, Hebei Normal University of Science & Technology, Qinghuangdao, China; ^4^College of Animal Science and Technology, Agricultural University of Hebei, Baoding, China; ^5^Kunming Institute of Zoology, Chinese Academy of Sciences, Kunming, China; ^6^Key Lab of Agricultural Animal Genetics, Breeding and Reproduction of Ministry of Education, College of Animal Science and Technology, Huazhong Agricultural University, Wuhan, China; ^7^Department of Herbivore Science, Yunnan Animal Science and Veterinary Institute, Kunming, China; ^8^Institute of Animal Science, Chinese Academy of Agricultural Sciences (CAAS), Beijing, China

**Keywords:** intersexuality, genome-wide selection, Hi-C, copy number variant, translocation

## Abstract

**Background:** Polled intersex syndrome (PIS) leads to reproductive disorders in goats and exerts a heavy influence on goat breeding. Since 2001, the core variant of an 11.7 kb deletion at ~129 Mb on chromosome 1 (CHI1) has been widely used as a genetic diagnostic criterion. In 2020, a ~0.48 Mb insertion within the PIS deletion was identified by sequencing in XX intersex goats. However, the suitability of this variation for the diagnosis of intersex goats worldwide and its further molecular genetic mechanism need to be clarified.

**Results:** The whole-genome selective sweep of intersex goats from China was performed with whole-genome next-generation sequencing technology for large sample populations and a case–control study on interbreeds. A series of candidate genes related to the goat intersexuality phenotype were found. We further confirmed that a ~0.48 Mb duplicated fragment (including *ERG* and *KCNJ15*) downstream of the ~20 Mb PIS region was reversely inserted into the PIS locus in intersex Chinese goats and was consistent with that in European Saanen and Valais black-necked goats. High-throughput chromosome conformation capture (Hi-C) technology was then used to compare the 3D structures of the PIS variant neighborhood in CHI1 between intersex and non-intersex goats. A newly found structure was validated as an intrachromosomal rearrangement. This inserted duplication changed the original spatial structure of goat CHI1 and caused the appearance of several specific loop structures in the adjacent ~20 kb downstream region of *FOXL2*.

**Conclusions:** Results suggested that the novel complex PIS variant genome was sufficient as a broad-spectrum clinical diagnostic marker of XX intersexuality in goats from Europe and China. A series of private dense loop structures caused by segment insertion into the PIS deletion might affect the expression of *FOXL2* or other neighboring novel candidate genes. However, these structures require further in-depth molecular biological experimental verification. In general, this study provided new insights for future research on the molecular genetic mechanism underlying female-to-male sex reversal in goats.

## Background

In as early as the nineteenth century, people regarded hornlessness as a beneficial and important economic trait and bred specialized hornless goat strains. However, during breeding, the proportion of intersex individuals in the hornless goat population gradually increased. This phenomenon was termed polled intersex syndrome (PIS) (Eaton, 1945). Intersexuality, the phenomenon wherein certain dioecious organisms possess both sexes, has been widely observed in various livestock species (Bosu and Basrur, 1984; Wang and Zhang, 1993), including goats (Ramadan and El Hassan, 1988; Ramadan et al., 1991), within the last century. The proportion of intersex goats within the global population is 4–15% (Zhan et al., 1994; Chen et al., 2010; Song et al., 2015). Reproductive system malformations in PIS goats lead to the loss of reproductive capacity and are thus some of the great challenges encountered in the development of the goat industry.

In 1996, the CHI1q41–q45 genomic regions were confirmed to be linked to hornlessness (Vaiman et al., 1996, 1999). Various molecular methods, such as chromosome walking technology and sequencing, have been used to refine the PIS locus to <100 Kb (Schibler et al., 2000). In 2001, the indicator of PIS in goats was mapped and resolved to an 11.7 kb non-coding deletion in CHI1q43 that was located ~200 kb upstream of the *FOXL2* gene (Pailhoux et al., 2001). *FOXL2* is an important sex determination gene (Bagheri-Fam et al., 2017; Tao et al., 2018) with a key role in ovarian development (Pannetier et al., 2016; Elzaiat et al., 2017). For example, a previous study on mouse models with ovarian *FOXL2* gene deletion showed that *FOXL2*^+/−^ mice have a normal phenotype, *FOXL2*^+/+^ mice have a similar phenotype as patients with human blepharophimosis syndrome, and *FOXL2*^−/−^ mice exhibit narrow eye slits and premature ovarian failure (Baron et al., 2005). Furthermore, by using gene editing technology, Boulanger et al. (2014) verified that the loss of function and expression silencing of *FOXL2* can cause female-to-male sex reversal in XX goats (Boulanger et al., 2014).

Moreover, the development of PIS diagnostic molecular markers can effectively avoid the misdiagnosis caused by the phenotypic identification of obscure PIS cases. Although a series of PIS diagnostic methods based on PCR amplification has been reported (Yang et al., 2012; Zhang et al., 2019), some studies on the diversification of PIS deletion structure have questioned the accuracy of these methods (Li et al., 2011; Kijas et al., 2013). For example, some intersex Rangeland goats do not exhibit the known homozygous PIS deletion (Kijas et al., 2013). Therefore, whether PIS deletion is specific for the diagnostics of intersexuality in goats remains controversial. Notably, the long-read whole-genome sequencing of two (one Saanen and one Valais Blacknecked black goats) genetically female (XX) intersex goats (Simon et al., 2020) demonstrated that a highly complex structural variant involving a ~0.48 Mb duplicated segment from ~21 Mb of chromosome 1 (CHI1) is inversely inserted into the known PIS deletion and that the length of the PIS deletion has also been shortened to 10.159 kb from 11.7 kb (Pailhoux et al., 2001).

In this study, we, for the first time, identified the intersex-related genetic variation structure of the Chinese goat population via high-throughput sequencing technology and analyzed the chromosomal spatial structure of the PIS-related genetic structure through high-throughput chromosome conformation capture (Hi-C) technology to obtain an in-depth understanding of the molecular genetic mechanism of PIS. Our work could also provide a valuable reference for the future development of diagnostic tools with enhanced broad-spectrum recognition capabilities.

## Methods

### Genomic Library Construction and Sequencing

All the experimental conditions of this study were approved by the Committee on the Ethics of Animal Experiments of the Southwest University (No. [2007] 3) and the Animal Protection Law of China.

We collected venous blood samples from 55 goats comprising 35 intersex goats (26 XX Tangshan dairy goats and 9 XX Chinese southern native goats) and 20 XX non-intersex Tangshan dairy goats. A total of 2 mL venous blood was collected from each animal (Sampling from Tangshan dairy goat breeding farm, Tangshan, China). The wound was sterilized with 70% medical alcohol. All 55 animals were returned to the pasture to continue living after experimentation. All genomic DNA samples were extracted by using a QIAGEN DNeasy Blood & Tissue kit in accordance with the manufacturer's protocol. Sequencing libraries were constructed with DNA extracts and a NEBNext® Ltra DNA library preparation kit (Illumina®, US). Sequencing was performed on an Illumina HiSeq × Ten platform (pair-end 150 bp). The sequencing data generated in this study were deposited in the NCBI SRA database (SRR10051499-SRR10551533 and SRR10613872-SRR10613891). In addition, we downloaded 166 non-intersex goat genome sequences from the NCBI SRA database. The detailed information of the 221 animals used in this study is shown in Supplementary Table 1.

### Read Filtering, Read Alignment, and Variant Calling

Raw sequencing reads were trimmed and filtered by using Trimmomatic (version 0.36). We then mapped the clean pair-end reads to a goat (*Capra hircus*) reference genome (ARS1) by using BWA-MEM (version 0.7.13) with default parameters except that “-M” was enabled to flag shortened split hits as secondary data. We used Picard (version 2.1.1, http://broadinstitute.github.io/picard) to remove potential PCR duplicates. Finally, the reads were locally realigned around indels with the IndelRealigner procedure in GATK (version 3.7). We applied GATK to call variants and used the HaplotypeCaller algorithm in Genomic Variant Call Format (GVCF) mode. Variants were called individually for each animal, and one GVCF file that listed genotype likelihoods was generated per animal. Then, the variants were called from the GVCF files through joint genotyping analysis. We removed SNPs that were within the three base pairs of an indel by utilizing bcftools (version 1.8). Biallelic SNPs were retained by applying a hard filter of QD < 2.0, MQ < 40.0, FS > 60.0, SOR > 3.0, MQRankSum < −12.5, or ReadPosRankSum < −8.0. We also used vcftools (version 0.1.14) to remove SNPs with a missing rating of more than 0.1. The copy number variations (CNVs) with a silhouette score of <0.65 and a MAF of <0.05 were identified by using CNVcaller software (Wang et al., 2017a).

### Genome-Wide Selective Sweep Analysis and Gene Annotation

Here, we carried out whole-genome selection signal analysis with two groups: (1) 35 intersex goats (case group, including 26 intersex Tangshan dairy goats [X/X] and 9 intersex Chinese goats [X/X] from southern China) vs. 186 non-intersex individuals (control group) and (2) 26 intersex goats (case group) vs. 20 non-intersex individuals (X/X, control group) from the Tangshan dairy goat population. For the SNP dataset, we calculated the pairwise fixation index (*F*_ST_) and π ratio (π_intersex_/π_non−intersex_) with 40 kb sliding windows and 10 kb step size. Only windows passing the above two thresholds were retained. Candidate genes were subjected to functional enrichment with an online tool (KEGG, http://www.genome.jp/kegg/pathway.html).

Additionally, we calculated *V*_*ST*_ and *F*_*ST*_ on the basis of absolute copy number (CN) to identify divergent CNV profiles between XX intersex and normal female goats. *V*_*ST*_ was calculated by using following formula: $V_{ST}=\frac{V_{total}\;-\;\left(V_{pop1\;}\times \;N_{pop1}\;+\;V_{pop2}\;\times \;N_{pop2}\right)/N_{total}}{V_{total}}$, where *V*_*total*_ is the total variance, *V*_*pop*_ is the CN variance for each population, *N*_*pop*_ is the sample size for each population, and *N*_*total*_ is the total sample size.

Lastly, we calculated linkage disequilibrium by using Arlequin software version 3.5.1.3 (Excoffier and Lischer, 2010) with a permutation test (EM algorithm, permutation number = 100,000).

### PCR Amplification to Verify Structural Variant Genotypes

The primers (Supplementary Table 2) of breakpoints based on the ~0.48 Mb fragment (CHI1:150,334,286–150,818,099) that was reversely inserted into the PIS deletion region (~10.16 kb, CHI1:129,424,780–129,434,939) were designed with the online tool Primer 3.04 software (http://bioinfo.ut.ee/primer3-0.4.0/) to identify the structural characteristics of the identified duplication variant in CHI1 in intersex goats. 2 × TransTaq® High Fidelity (HiFi) PCR SuperMix II (TransGen Biotech, China) was used in PCR amplification. The qPCR reaction conditions consisted of an initial denaturation at 94°C for 5 min, followed by 35 cycles of denaturation at 94°C for 30 s, annealing at the locus-specific temperatures presented in Supplementary Table 2 for 30 s, and extension at 72°C for 120 s. Finally, an elongation step (final extension) was performed at 72°C for 7 min. Two-way Sanger sequencing was performed on an ABI 3730 sequencer platform (Life Technologies, US).

### Three-Dimensional Genome Structure Comparison Between Intersex and Non-intersex Goats

Hi-C was performed on two individuals (Dazu black goat, China). One was intersex (hornless, PIS +/+), and the other was non-intersex (horned, female, PIS –/–). Sample processing (treatment of leukocytes from venous blood with the cell cross linker paraformaldehyde) and library construction were performed by using standard methods (four cutter restriction enzyme [MboI], Belton et al., 2012). Briefly as: (1) treat cells with paraformaldehyde (37% formaldehyde) to fix the conformation of DNA; (2) treat cross linked DNA with restriction enzymes (four cutter restriction enzyme, MboI) to produce sticky ends; (3) repair DNA ends with biotin labeling; (4) connect the DNA fragments by DNA ligase; (5) release the cross linked DNA state with 2.5M Glycine; (6) purify the DNA by AMPure XP system (Beckman Coulter, Beverly, USA) and randomly break into 300~500 bp fragments; (7) construction of small DNA fragment library using NEB Next Ultra DNA Library Prep Kit (NEB, USA). After library construction, Qubit2.0 was used for preliminary quantification. Then, the library was diluted to 1 ng /μL. Agilent 2100 was used to determine whether the insert size of the library met expectations. Q-PCR was used to quantify accurately the effective concentration of the library (>2 nM), and sequencing was finally performed with an Illumina HiSeq × Ten PE150^TM^ platform. Sequencing data quality control, reference genome alignment (ARS1), interaction map construction, and loop structure analysis were performed with Juicer software (Durand et al., 2016) with the standard parameters (Mbol restriction enzyme chunk size set at: 80000000 bp). Image visualization was performed by using the matplotlib package in the Python environment.

## Results

**Figure 1:**
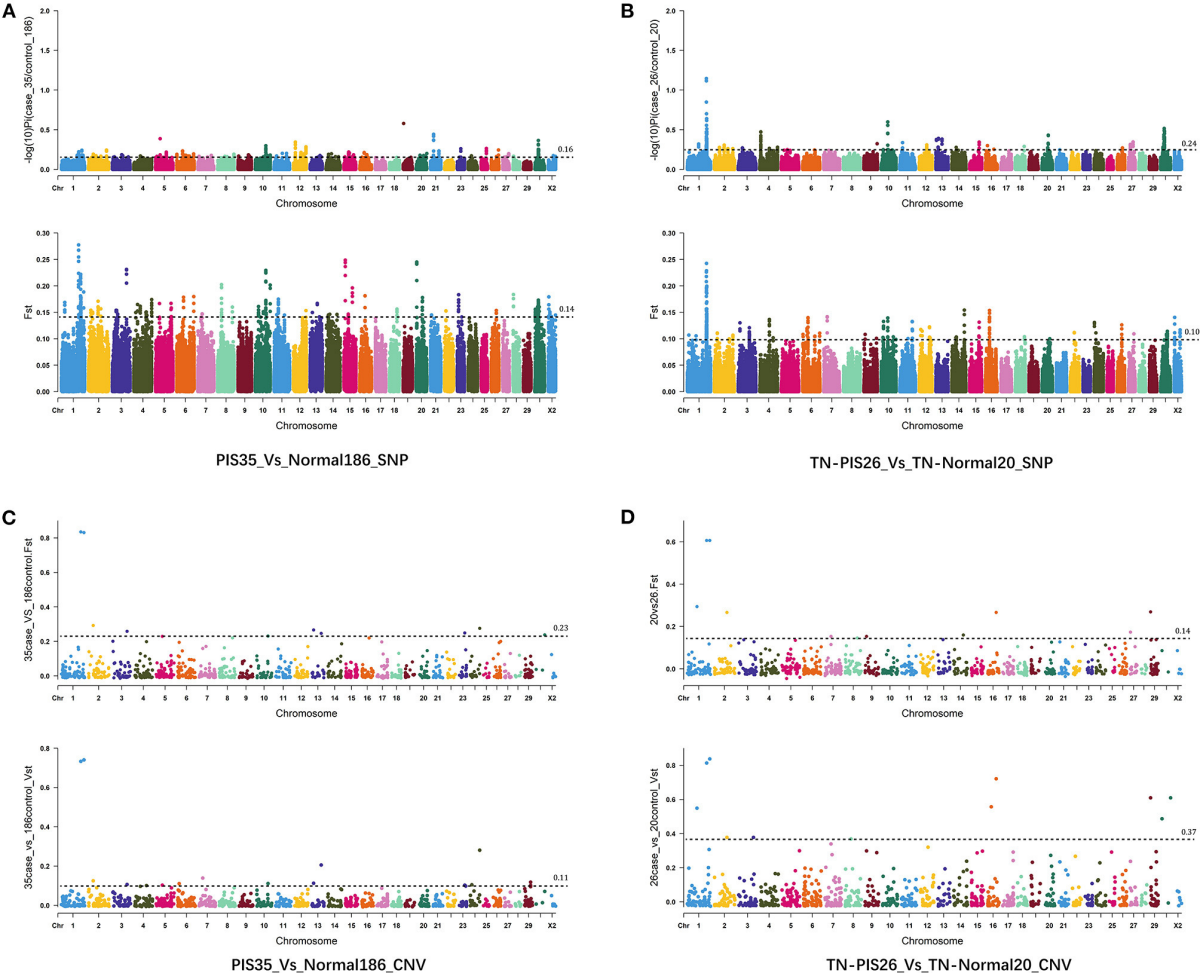
Genome-wide selective sweep of goat intersexuality by using SNPs and CNVs. **(A)** Manhattan plot showing the SNP-based selection signals of 35 intersex goats compared with those of 186 non-intersex goats from a large geographically distributed population. **(B)** Manhattan plot showing the SNP-based selection signal of intersex goats within the Tangshan dairy goat population. **(C)** Manhattan plot showing the CNV-based selection signals of 35 intersex goats compared with those of 186 non-intersex goats from a large geographically distributed population. **(D)** Manhattan plot showing the CNV-based selection signals of intersex goats within the Tangshan dairy goat population.

A total of 16 462,769 SNPs and 1,058 CNVs were obtained from 221 samples. For the genome-wide selection of SNPs in all individuals (35 intersex vs. 186 non-intersex goats), we screened 258,064 windows and estimated their *F*_*ST*_ and π ratios (Figure 1A, Supplementary Table 3). In total, we identified 40 windows in accordance with the intersection of the top 1% selective regions of both parameters (F_ST_ and π ratio). These regions included 74 coding genes, which encompassed or were located up- and downstream within the 300 kb range of the window. Six of these genes were annotated to seven known signaling pathways (Supplementary Table 4), including neuroactive ligand-receptor interaction (*P2RY13* and *P2RY14*), hippo signaling pathway-multiple species (*STK3*), thyroid hormone signaling pathway (*MED12L*), hippo signaling pathway (*STK3*), *MTOR* signaling pathway (*RRAGB*), protein processing in endoplasmic reticulum (*UBQLN2*), and *MAPK* signaling pathway (*STK3*).

Furthermore, we selected 46 Tangshan dairy goats from large samples, set up a scientific case–control analysis test, and identified the selected signal regions of intersexuality within Tangshan dairy goat populations to prevent the genetic background divergence of large sample populations from interfering with selection signal analysis (Figure 1B, Supplementary Table 5). The results revealed that 50 windows were generated by the intersection of the top 1% selective regions of the *F*_*ST*_ and π ratios and 79 genes, which covered or were located up- and downstream within the 300 kb range of the window. Only four genes were enriched in known pathways (Supplementary Table 6). These genes included *RBP2* (vitamin digestion and absorption), *NCK1* (*ErbB* signaling pathway, T cell receptor signaling pathway, and Axon guidance), *IL20RB* (Jak-*STAT* signaling pathway and cytokine–cytokine receptor interaction), and *MRAS* (tight junction, phospholipase D signaling pathway, proteoglycans in cancer, *Rap1* signaling pathway, regulation of actin cytoskeleton, *Ras* and *MAPK* signaling pathways, and HTLV-I infection). Numerous consecutive windows in CHI1 (~129 to ~132 Mb) of intersex Tangshan dairy goats showed strong *F*_*ST*_ and π ratio signals, and these windows covered the 11.7 kb fragment deletion (PIS) that is widely recognized genomic signature of XX intersex goats (Pailhoux et al., 2001).

The selective CNV-based sweep analysis of intersexuality with a large population and various genetic backgrounds of non-intersex goats (35 vs. 186) revealed that two CNVs had the highest signals with *F*_*ST*_ (V1: *F*_*ST*_ = 0.834565, CHI1:129,424,780–129,434,939; V2: *F*_*ST*_ = 0.830614, CHI1:150,334,286–150,818,099) and *V*_*ST*_ (V1–V_ST_ = 0.73290; V2–*V*_*ST*_ = 0.74050) (Figure 1C, Supplementary Table 5). These two variants also carried the most prominent signal in the Tangshan dairy goat population (20 vs. 26; V1: *F*_*ST*_ = 0.60641, *V*_*ST*_ = 0.81321; V2: *F*_*ST*_ = 0.60641, *V*_*ST*_ = 0.83742; Figure 1D, Supplementary Table 7). The V1 variant was contained within the known intersex-related variant region (PIS deletion). Two genes, namely, *MRPS22* (~120 kb distance) and *COBB2* (~140 kb distance), were found upstream of V1, whereas the *FOXL2* gene was found 340 kb downstream of V1. Other seven noncoding RNA (*LOC102190268, LOC108636915, LOC102185085, LOC108636375, LOC102190822, LOC102191084*, and *LOC100861210*) were discovered between *FOXL2* and V1. Furthermore, *KCNJ15* and *ERG* were encompassed by the V2 variant region, and *ETS2* was located 230 kb downstream. No coding gene was found within the 500 kb upstream region of V2.

**Figure 2:**
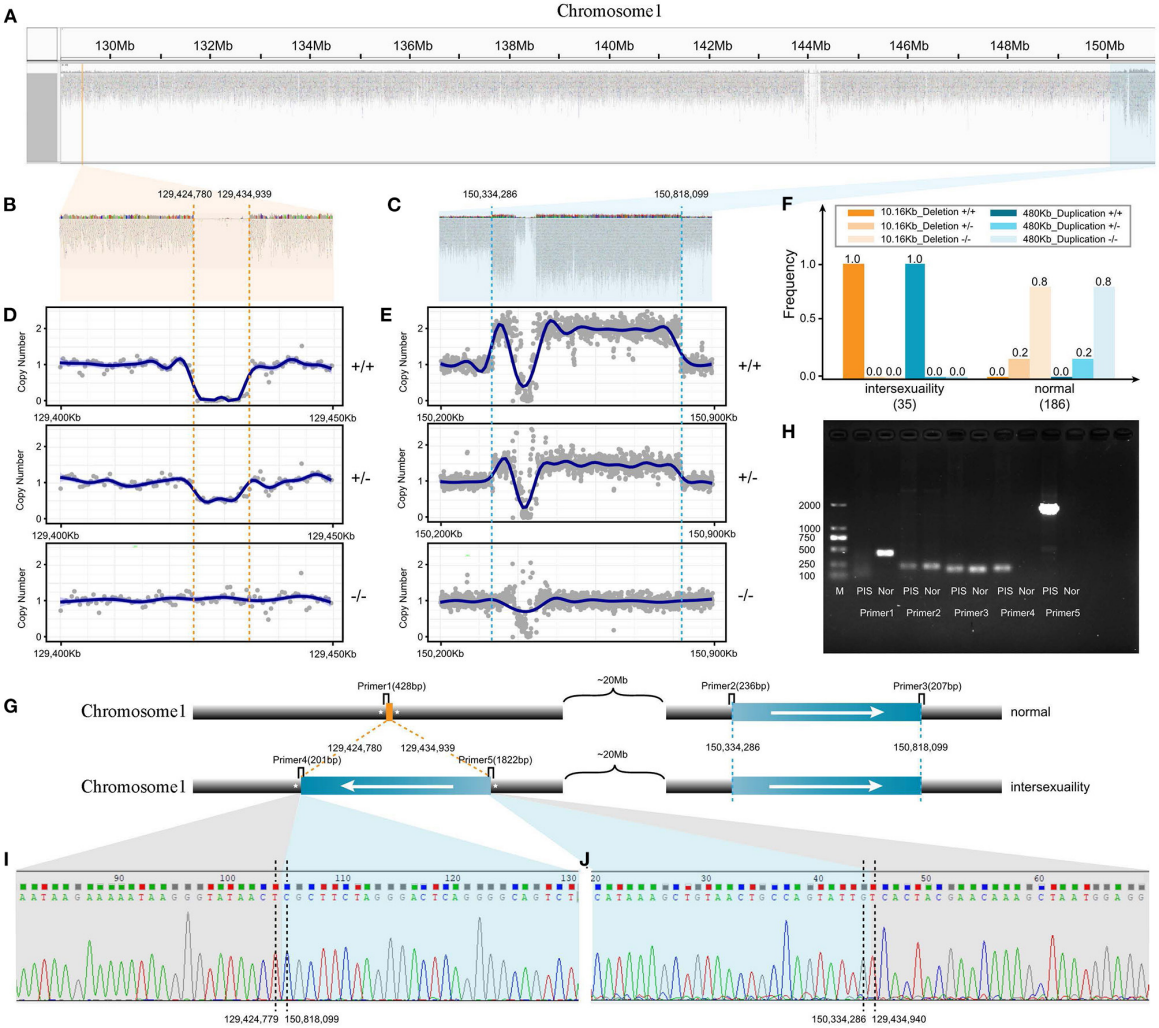
Model detection and verification of novel transposition in intersex goats. **(A)** Two CNVs (V1 and V2) on Chromosome1 observed by IGV software. **(B)** Alignment and coverage of wide-genome short-reads from intersex goats as observed with IGV software. The CNV from 129,424,780 to 129,434,939 bp on Chromosome 1 (V1, **B**) manifested as a deletion. **(C)** Alignment and coverage of wide-genome short-reads from intersex goats as observed by IGV software. The CNV from 150,334,286 to 150,818,099 bp on Chromosome 1 manifested as a duplication (V2). **(D)** Genomic coverage of different genotypes of V1 variant reads (Chromosome 1, 129.40–129.45 Mb). **(E)** Genomic coverage of different genotype of V2 variant reads (Chromosome 1, 150.20–150.90 Mb). **(F)** Two CNV variants (V1 and V2) associated with intersexuality had the same frequency in the population. **(G)** Schematic of the Chromosome 1 PIS transposition model and location map of primers for PCR verification. **(H)** Gel electrophoresis verification of PCR results. **(I,J)** Sanger sequencing results of sequences amplified with primers 4 and 5.

The length of the PIS deletion (V1) on CHI1 was ~10.16 kb and was located from 129,424,780 bp to 129,434,939 bp (Figures 2A,B) as observed by using the IGV browser (Thorvaldsdóttir et al., 2013). We found that the length of the V2 variant was ~0.48 Mb and that this variant was distributed on CHI1 at 150,334,286–150,818,099 (Figure 2C). The different genotypes of the V1 and V2 variant regions could be clearly identified by comparing the read average coverage of each variant's region with that of the whole genome (Figures 2D,E). On the basis of the genotypes of V1 and V2, we found that the homozygous deletion of V1 and the homozygous duplication of a ~0.48 Mb region of V2 were always simultaneously present in all intersex goats (Figure 2F). Linkage disequilibrium analysis revealed significant linkage (*P* < 0.0001) between the V1 and V2 mutations in 221 goats.

In the heterozygous and extra duplication homozygous individuals with the V2 mutation, a considerable number of reads were split-mapped simultaneously to the outer boundary of V1 and the inner boundary of V2 (Supplementary Figure 1). We verified the true boundary breakpoints of the two variant regions through PCR amplification and Sanger sequencing (Figures 2G–J). Therefore, the precise PIS genome structure was doubly confirmed as an inverted duplication of the ~0.48 Mb segment that had inserted into the 10.16 kb PIS deletion.

An average of >250 Gb (~85×) of genome coverage data were obtained from two individuals and used to construct a 3D genome high-resolution interaction map. Firstly, we gathered total 851,483,595 and 808,343,466 reads in case and control individual, respectively. Secondly, according to the mapping results of case and control dataset, there are 300,895,514 (35.34%) and 359,770,248 (44.51%) normal paired reads, 402,929,844 (47.32%) and 347,404,121 (42.98%) chimeric paired reads, 344,755,346 and 117,458,553 PCR duplicates reads, 156,341,608 and 244,912,442 intra-chromosomal interaction reads, 71,982,908 and 173,316,912 short-distance interaction sequence with interaction distance less than 20 kb (<20 kb), 84,358,664 and 149,510,017 long-range interaction sequences with interaction distance larger than 20 kb (>20 kb), respectively.

**Figure 3:**
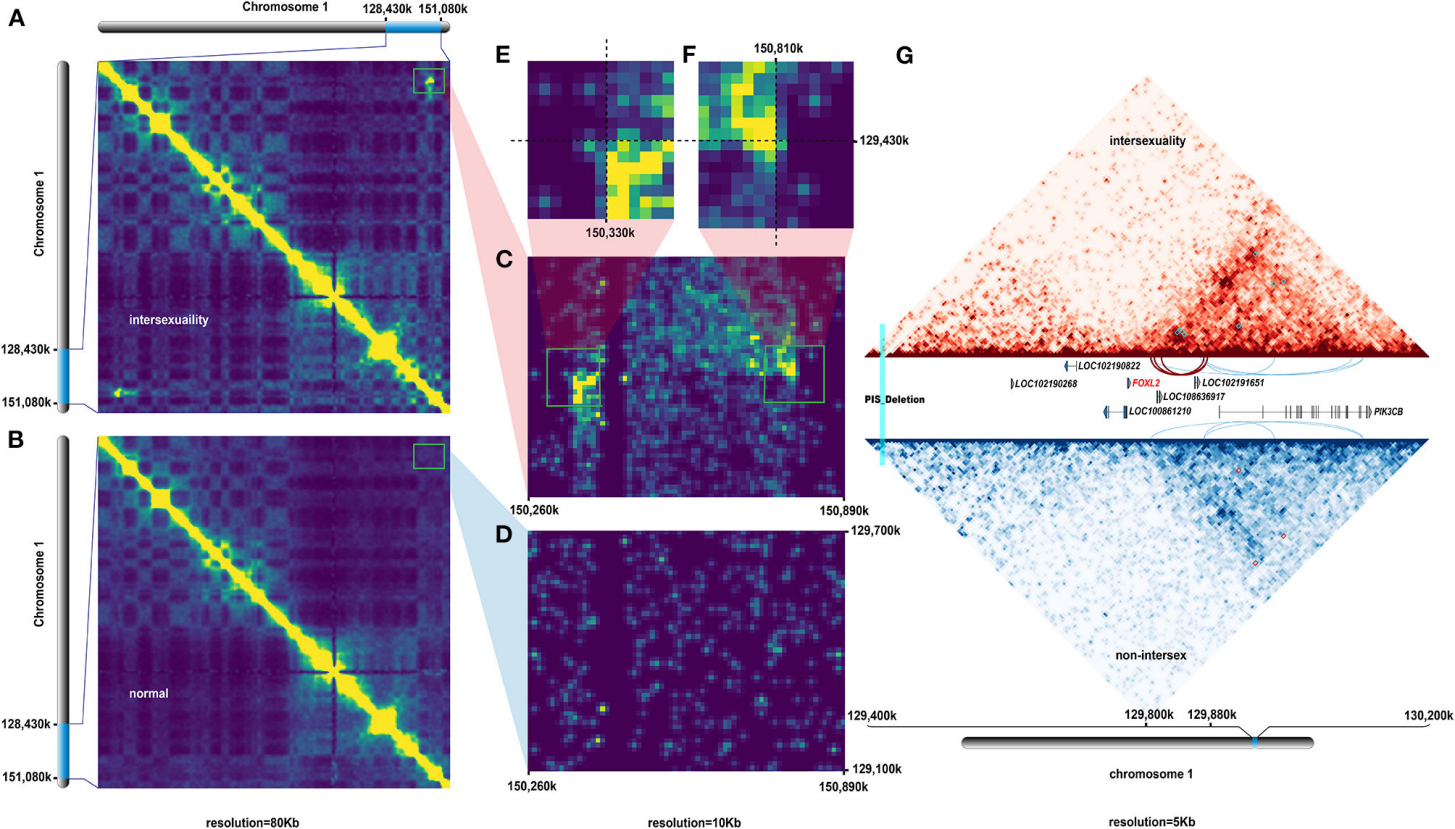
Hi-C analysis results. **(A)** Window interaction matrix on CHI1 from 128.43 to 151.08 Mb in intersexuality individuals with 80 Kb resolution. **(B)** Window interaction matrix on CHI1 from 128.43 to 151.08 Mb in normal control individuals with 80 Kb resolution. **(C–F)** Interaction matrix and comparison of the 129.1–129.7 and 150.3–150.9 Mb regions on CHI1 between intersexuality and normal individuals with 10 Kb resolution. **(G)** Analysis of loop conformation in the 129.4–130.2 Mb region of CHI1 between intersexuality and normal individuals at 5 kb resolution revealed that the intersexuality case had a particular loop conformation in the 129.80–129.88 Mb region at which the sexual development-associated gene *FOXL2* was located 30 Kb upstream.

The heat map of both sets of data with 80 kb resolution revealed a potential intrachromosomal rearrangement site (Figures 3A,B), which was initially identified in CHI1 of an intersex individual (PIS–/–). This finding was consistent with the physical location of V2, which was absent from non-intersex goats (PIS–/–). We used a resolution of 10 kb to identify effective breakpoints (Figures 3C,D). A small but sharp contact peak suggested that a new intrachromosomal rearrangement occurred in CHI1 of the intersex goats (Figures 3E,F). We identified four private consequent loop regions in CHI1 of the intersex goats (Supplementary Table 8) and compared these regions with those in non-intersex individuals (Supplementary Table 9). These loop regions were densely clustered in the ~20 kb downstream regions of the *FOXL2* gene, which overlapped with LOC102191651 and LOC108636917 (Figure 3G).

## Discussion

SNP-based genome-wide selection signal analysis revealed numerous sharp signals in 35 intersex goats and 186 control samples. Within the top 1% selection window, a series of genes were identified and found to be deeply involved in animal reproduction and multiple developmental processes. For example, *STK3* is a key molecule that connects the downstream signaling pathway of estrogen and the Hippo signaling pathway; it also regulates the dynamic development of the uterine epithelium during the estrous cycle through the signal transduction of uterine epithelial cells (Moon et al., 2019). *STK3* was annotated to other signaling pathways, such as the *MAPK* (Bogani et al., 2009; Warr et al., 2016) and Hippo signaling pathways, that also play an important role in gonadal development and sex determination (Frum et al., 2018; Devos et al., 2020). Although *MED12L* has been verified to be associated with fetal mental retardation in human (Nizon et al., 2019), it is also involved in reproductive development (Sayem et al., 2017; Das and Kumar, 2018; Ulloa-Aguirre et al., 2018). In addition, the *RRAGB* gene is enriched in the *mTOR* signaling pathway, which is widely involved in gonadal development (Bajwa et al., 2017; Correia et al., 2020). Therefore, our findings suggested that numerous molecular mechanisms underlying development and the physiological maintenance of intersexual characteristics await further excavation.

Notably, the different genetic backgrounds of large samples can cause many false-positive genes, and chromosomal regions may thus be incorrectly identified. Given the inconsistent ratios between the numbers of Tangshan dairy goats in the intersex and control groups, a gene with considerable breed specificity caused interference. We performed a strict case–control experiment on the Tangshan dairy goat population to prevent the interference of specific population backgrounds and identified a series of interesting genes. The highest continuous selection signal was observed in CHI1. These signals covered the areas of a previously reported PIS deletion (Pailhoux et al., 2001) and six coding genes (*MRAS, NMNAT3, ARMC8, DBR1, LOC108636376*, and *SCLC35G2*); *SOX14* and *MRPS22* in the upstream region; and *LOCl02190268* and *IL20RB* in the downstream region.

Some genes with cellular biological importance were identified. For example, *NMNAT3* maintains cell differentiation by maintaining mitochondrial content (Son et al., 2016; Yu et al., 2020). *ARMC8* is involved in the adherence of regulatory cells to cells and is associated with cell differentiation in ovarian cancer tumors (Jiang et al., 2015; Gul et al., 2019). *SOX14* is associated with apoptosis in cancer cells in the sexual reproductive system (Stanisavljevic et al., 2017) and is a crucial determinant of allergy development in *Drosophila* (Ritter and Beckstead, 2010). Interestingly, the conserved region of the *MRPS22* gene is a long-range enhancer and regulates the expression of *FOXL2* through an unclear advanced *cis*-regulatory effect of chromatin structure in humans and rats (Crisponi et al., 2004). Thus, our case–control experiment involving a population with a single genetic background enabled us to screen out many false-positive signals and identify a series of credible candidate genes. The results of this experiment provided insight into the molecular genetic mechanism of intersexuality-related physiology.

The recent identification of the gene transcription profiles of intersex and normal goat gonads through the use of RNA-Seq technology suggests that a large number of differentially expressed genes may be involved in the regulation of sex determination and differentiation in intersex goats (Yang et al., 2020). This result reminds us that many potential molecular mechanisms under the goat sexual reversal phenotype remain unclear.

Our CNV-based analysis results showed that equally strong signals were generated in V1 and V2 in the large sample with different genetic backgrounds and the Tangshan dairy goat population in the case–control analysis. These signals were recognized as the 10.16 kb PIS deletion and the ~0.4838 Mb duplicated segment located ~20.9 Mb further downstream of the PIS deletion and ~150 Mb on CHI1. In addition, as expected, the highly complex structure was identified as the additional 0.4838 Mb-sized duplicated segment that was inversely inserted at the breakpoint of the 10.16 kb deletion. Our findings were consistent with the recent research results from a team in Germany that used long-read whole-genome sequencing (Simon et al., 2020). Although we utilized short-read sequencing technology, the large sample size and classic case–control experimental design still achieved the same effect. Our study confirmed that the XX intersex goats from the hornless goat population in China share the same PIS genome variant structure with European goats.

In accordance with previous studies that identified the segment size and polymorphism in PIS deletion (Li et al., 2011). We believe that some loss in the ASR1 assembly occurred on the last 180 bp section, while was not lost on the previous 11.7 kb PIS deletion sequence (GenBank No. AF404302) investigated by Simon et al. (2020). It was adjacent to the *PISRT1* gene with the closest physical distance. However, previous studies have shown that *PISRT1* does not participate in the expression of *FOXL2* and the determination/differentiation of the gonads. For example, the overexpression of *PISRT1* in PIS–/– fetuses does not affect *FOXL2* expression levels and gonadal development (Boulanger et al., 2008).

The duplicated segment contained the *KCNJ15* and *ERG* genes. The extra copies of these two genes have an essential role in horn and gonadal development. *KCNJ15* is known to participate in insulin secretion (Okamoto et al., 2012), nervous system diseases (Zhou et al., 2018), gastric acid secretion (Yuan et al., 2015), kidney cancer (Liu et al., 2019), and esophageal squamous cell carcinoma (Nakamura et al., 2020). It is also involved in gastric acid secretion and regulation (He et al., 2011), and the relationship between gastric acid secretion and the effects of sex hormones was verified decades ago (Adeniyi, 1991). The high expression of *KCNJ15* in follicle-associated epithelium suggests that *KCNJ15* may be involved in the functional development of the ovary (Kobayashi et al., 2012) and implicates this gene in female gonadal development. Furthermore, a large number of studies have shown that *ERG* is not only an oncogene that is related to a variety of cancers (Wang et al., 2017b; Zhang et al., 2020), it also participates in the embryonic developmental processes, including bone development (Iwamoto et al., 2000), of a variety of organisms (Furlan et al., 2005; Nikolova-Krstevski et al., 2009). This participation indicates that the *ERG* gene may be related to horn and embryonic development.

Furthermore, Hi-C technology was used to study DNA replication, transcription regulation, and DNA damage repair and contact between chromosomal loci (Cremer and Cremer, 2001; de Wit and De Laat, 2012; Maass et al., 2018). Currently, this topic is heavily explored in genomic research, and numerous studies on technical method optimization have been performed (Lin et al., 2018; Yardimci et al., 2019; Janaratne et al., 2020).

Intrachromosomal rearrangement or palindrome duplication is associated with various processes of phenotypic determination and development (Carbonell-Bejerano et al., 2017; Yin et al., 2017; Mendoza et al., 2020). We performed the loop analysis of the 3D genomes to further investigate the special chromosomal spatial structures resulting from the identified intrachromosomal rearrangement. We found several unique loop structures in CHI1 of homozygous PIS intersex goats but not in that of non-intersex individuals. Many intrachromosomal rearrangement structures can alter gene expression levels within and in areas adjacent to a gene region by altering chromosomal structure (Demura et al., 2007; Suzuki et al., 2020).

Substantial evidence indicates that many of the observed loops are related to gene regulation and serve as anchors and promoters (Ahmadiyeh et al., 2010; Hoffman et al., 2013; Rao et al., 2014). The loops that we identified in this study were consistent and clustered near the *FOXL2* gene. Numerous pieces of evidence have shown that silencing *FOXL2* expression directly affects ovarian development and oogenesis in fish (Fan et al., 2019), mice, and humans (Uda et al., 2004; Thanatsis et al., 2019). Specifically, the elimination of *FOXL2* expression is sufficient to induce female-to-male reversal in XX goats (Pannetier et al., 2012; Boulanger et al., 2014). Therefore, although the regulatory relationship between this newly discovered intrachromosomal rearrangement and *FOXL2* expression cannot be evaluated thus far, the change in spatial chromosome 3D structure in the adjacent region of *FOXL2* was evident. Whether these loop structures affect *FOXL2* expression and cause intersexuality by inhibiting *cis*-acting elements or switching *trans*-acting elements should be evaluated through in-depth molecular biology research.

In addition, we found that two genes were located within the loop region: one was trafficking protein particle complex subunit 1 pseudogene (LOC102191651), and the other was an uncharacterized non-coding RNA (LOC108636917). Additional evidence regarding the further functions of these both genes remains lacking. Therefore, we cannot conclude that these loops/two genes participate in gonadal development. However, an interesting gene, *PIK3CB*, that was located further downstream of LOC102191651 and LOC108636917 attracted our attention. Numerous studies have shown that *PIK3CB* plays an important role in the development and physiological function of the ovary (Zheng et al., 2012; Li et al., 2013; Nteeba et al., 2017). However, supernumerary data suggesting that this gene is responsible for the occurrence and maintenance of the intersexual phenotype are unavailable. Therefore, whether the novel loop region containing both genes affects *PIK3CB* expression and whether *PIK3CB* is a new essential factor that is sufficient for causing female-to-male sex reversal in XX goats need to be evaluated.

## Conclusions

We performed the genome-wide selective sweep of intersex goats with wide-genome next-generation sequencing. We doubly verified that the structural variant of caprine PIS structure, a 0.48 Mb duplicated fragment located ~20 Mb downstream of the PIS region that was reversely inserted into the PIS deletion, was sufficient as a broad-spectrum clinical diagnostic marker of XX intersex goats from Europe and China. The existence of several private dense loop structures in the region adjacent to *FOXL2* of intersex XX goats but not in that of non-intersex individuals suggested that intrachromosomal rearrangement might affect the expression of *FOXL2* or other neighboring novel candidate genes. This effect needs to be further evaluated. This study supported a precise genomic feature of PIS phenotype in intersex goats from Europe and China and provided new insights for future research on the molecular genetic mechanism underlying female-to-male sex reversal in goats.

## Data Availability Statement

The datasets presented in this study can be found in online repositories. The names of the repository/repositories and accession number(s) can be found at: https://www.ncbi.nlm.nih.gov/genbank/, SRR10051499-SRR10551533; https://www.ncbi.nlm.nih.gov/genbank/, SRR10613872-SRR10613891.

## Ethics Statement

The animal study was reviewed and approved by Animal Experiments of the Southwest University (No. [2007] 3) Southwest University, Chongqing 400716, China.

## Author Contributions

G-XE, YJ, Y-FH, Y-JZ, Y-HM, J-HZ, Q-HH, M-XC, and X-LL conceived and designed the experiments. D-KZ, Z-QZ, B-GY, H-JG, Y-MH, and X-HD analyzed the data. G-XE analyzed the data and wrote the paper. YJ, X-PJ, R-SN, Y-GH, YZ, R-YZ, W-HN, and L-HL supported the samples and displayed lab work. G-XE, Y-FH, and YJ provided funding. All authors read and approved the manuscript.

## Conflict of Interest

The authors declare that the research was conducted in the absence of any commercial or financial relationships that could be construed as a potential conflict of interest.

## Abbreviations

PIS: Polled intersex syndrome
CNV: Copy number variant
FST: Pairwise fixation index
SNP: Single nucleotide polymorphism
Hi-C: High-through chromosome conformation capture
CHI: *Capra hircus* chromosome
*FOXL2*: Forkhead box L2
*KCNJ15*: Potassium inwardly rectifying channel subfamily J member 15
*PIK3CB*: Phosphatidylinositol-4,5-bisphosphate 3-kinase catalytic subunit beta.
