# Supplementary material for: Identification of a Goat Intersexuality-Associated Novel Variant Through Genome-Wide Resequencing and Hi-C
Source: Front Genet. 2021 Feb 9;11:616743. doi: 10.3389/fgene.2020.616743 (PMC7901718; doi:10.3389/fgene.2020.616743)
Supplement: Supplementary file 10 [file Image_1.pdf]

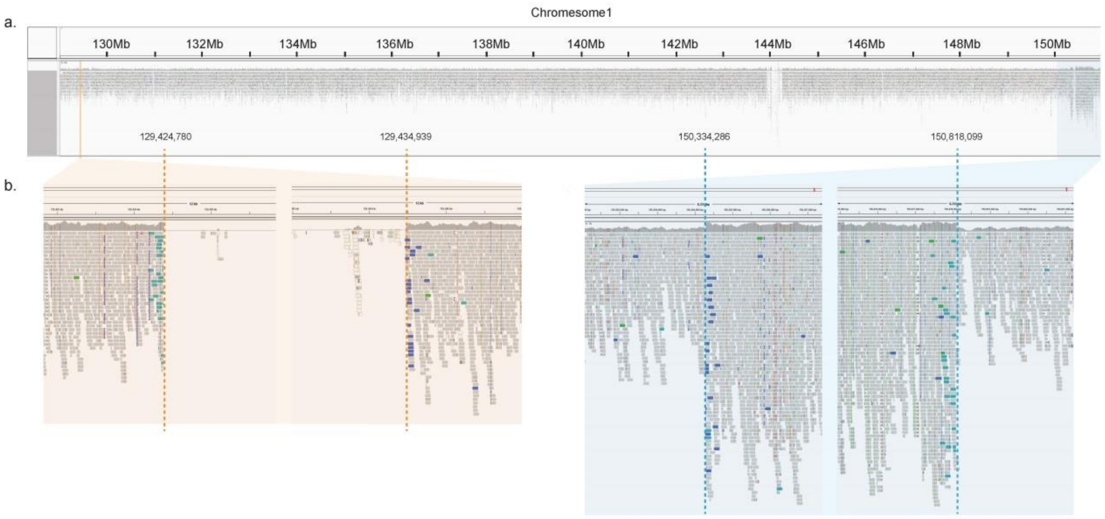

Figure S1 (a) IGV screenshots of the short-read sequence alignment showing a ~130 homozygous 10.1 kb deletion (yellow marker) and ~150 Mb homozygous ~0.48 Mb copy (bright blue marker). **(b)** Close-up of the IGV screenshot of the Illumina short-read sequence highlighted the deletion (V1) and copy variation (V2) and showed the boundary between the two variant sites (indicated by bright green and dark blue) with the existing amount of inconsistent paired end sequence reads arranged on two different variants on CHI1.
